# Microsatellite Instability Use in Mismatch Repair Gene Sequence Variant Classification

**DOI:** 10.3390/genes6020150

**Published:** 2015-03-30

**Authors:** Bryony A. Thompson, Amanda B. Spurdle

**Affiliations:** 1 Genetics and Computational Biology Department, QIMR Berghofer Medical Research Institute, 300 Herston Road, Brisbane, QLD 4006, Australia; E-Mail: Amanda.Spurdle@qimrberghofer.edu.au; 2 Department of Oncological Sciences, Huntsman Cancer Institute, University of Utah, 2000 Circle of Hope, Salt Lake City, UT 84112, USA

**Keywords:** lynch syndrome, variants of uncertain significance, mismatch repair, variant classification, multifactorial likelihood model

## Abstract

Inherited mutations in the DNA mismatch repair genes (MMR) can cause MMR deficiency and increased susceptibility to colorectal and endometrial cancer. Microsatellite instability (MSI) is the defining molecular signature of MMR deficiency. The clinical classification of identified MMR gene sequence variants has a direct impact on the management of patients and their families. For a significant proportion of cases sequence variants of uncertain clinical significance (also known as unclassified variants) are identified, constituting a challenge for genetic counselling and clinical management of families. The effect on protein function of these variants is difficult to interpret. The presence or absence of MSI in tumours can aid in determining the pathogenicity of associated unclassified MMR gene variants. However, there are some considerations that need to be taken into account when using MSI for variant interpretation. The use of MSI and other tumour characteristics in MMR gene sequence variant classification will be explored in this review.

## 1. Introduction

Identification of cancer-causing germline variants in high-risk cancer genes in cancer cases, directs the clinical management of not only the individual, but also the whole family. These measures include presymptomatic surveillance, prophylactic surgery and chemotherapy regimens [1]. Thus it is very important to identify carriers of pathogenic (disease causing) variants in these cancer genes.

Germline variants in the mismatch repair (MMR) genes (*MLH1*, *MSH2*, *MSH6*, and *PMS2*) that abrogate protein function cause the cancer susceptibility disorder, Lynch Syndrome [2]. Affected families often present clinically with multiple cases of early onset colorectal and endometrial cancers across several generations [3,4]. It is the most common hereditary form of these cancers, accounting for about 1%–3% [5,6,7,8]. Consistent with mutations in genes integral to DNA mismatch repair, the tumours develop through a mutator phenotype pathway, which is characterized by clonal global expansion or contraction of genomic short repetitive elements (microsatellites) producing the defining molecular signature of widespread somatic microsatellite instability (MSI) [9,10,11].

Identification of Lynch syndrome cases is very important, because these individuals have an increased risk of developing multiple metachronous and synchronous tumours [12,13]. Once an at-risk individual is identified for diagnostic testing, an algorithmic approach is generally used to detect MMR mutations. MSI testing and/or MMR protein immunohistochemistry (IHC) are used to identify MMR deficiency in tumours [14,15,16,17,18]. Somatic acquired alterations, such as *MLH1* promoter CpG island methylation and biallelic somatic mutations in the MMR genes are increasingly being used to identify so-called spontaneous MMR deficient colorectal and endometrial tumours [19,20,21,22,23,24,25]. Presence of the oncogenic *BRAF* V600E mutation in colorectal tumours can also be used to indicate spontaneous cases, but it is not a useful marker for triaging endometrial tumours [21,26]. The remaining cases are prioritised for genetic testing. When a pathogenic variant is identified in a case there are obvious clinical ramifications, so accurate variant classification is important. Tumour MSI status and other clinicopathological characteristics can aid in variant interpretation. Here, we describe the role MSI plays in MMR gene sequence variant classification.

## 2. MMR Gene Sequence Variant Classification

MMR gene sequence variants that are predicted to lead to premature termination of translation or a truncated protein are assumed to alter gene/protein function, and are thus classified as pathogenic (disease-causing). The remainder start as unclassified variants, as the effect on protein function of these variants is difficult to interpret. Such sequence variants include nucleotide changes predicted to cause missense substitutions, small in-frame insertions/deletions, or intragenic/intergenic sequence changes that may alter splicing or gene regulation. The issue of variants of uncertain clinical significance poses a major problem for clinicians, researchers and families that are suspected to have Lynch syndrome [1]. Recently, the International Society for Gastrointestinal Tumours (InSiGHT) has adopted a five-tiered variant classification scheme for interpretation of sequence variants identified in the four MMR genes [27]. The five classes are as follows: Class 5 (pathogenic), class 4 (likely pathogenic), class 3 (uncertain), class 2 (likely not pathogenic), and class 1 (not pathogenic). Pathogenicity is defined as being clinically relevant in a genetic counselling setting. The scheme incorporates qualitative or quantitative evidence to achieve sequence variant classification. Both approaches require multiple points of evidence, and are linked to clinical recommendations. The strength of the five-tiered scheme is its ability to utilize multiple sources of data readily available in the clinical setting.

### 2.1. Qualitative Classification Scheme

The InSiGHT classification rules include sequence-based criterion to annotate mutations that are widely accepted as pathogenic in standard clinical practice, *i.e*., nonsense/frameshift mutations and partial gene deletions. In contrast, for MMR variants of unknown clinical significance there are several criteria that can be assessed for qualitative classification, none of which should be used alone. These include analysis of tumour characteristics, such as MSI and IHC (loss of expression of the appropriate protein/s) [28,29,30], which could demonstrate an association of the variant with Lynch syndrome. Non-tumour characteristics associated with pathogenic mutations include, segregation with disease, absence in control individuals, and a deleterious effect in functional assays (protein function or splicing) [28,29,30,31,32]. Furthermore, co-occurrence *in trans* with a pathogenic mutation, without an abnormal phenotype (*i.e.*, constitutional MMR deficiency—CMMR-D [33]) also suggests that a variant is likely not pathogenic.

### 2.2. Multifactorial Likelihood Model

**Figure 1:**
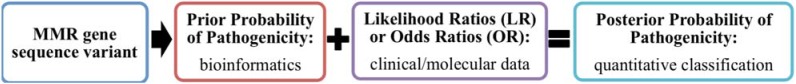
Outline of the mismatch repair genes (MMR) multifactorial likelihood model.

Multifactorial likelihood analysis was established to tackle the issue of unclassified variants in the familial breast cancer genes *BRCA1*/2 [34,35], and has since been applied to variants in *CDKN2A* [36] and the MMR genes [27,37]. It provides a quantitative measure of probability in favour of pathogenicity linked to clinical recommendations [38]. The model integrates different lines of genetic evidence using Bayesian analysis [34,39]. See an outline of the MMR multifactorial model in Figure 1. Each variant starts with a “prior probability” of pathogenicity, which can be based on *in silico* analyses. The approach used for the MMR genes to measure the prior probabilities for missense substitutions was to calibrate *in silico* algorithm outputs predicting the effects based on conservation and physicochemical properties of the residues [40,41], against a reference set of variants that were classified with confidence using the types of data mentioned above [42].

A “posterior probability” of pathogenicity is derived by updating the prior probability with likelihood ratios (LR) or odds ratios (OR) for pathogenicity determined from statistical analyses of observational data by comparing cases with known pathogenic variants to individuals with no pathogenic variants in a large dataset [34]. The posterior probability is used to classify a sequence variant based on the cut-offs for the classes (5, >0.99; 4, 0.95–0.99; 3, 0.05–0.949; 2, 0.001–0.049; 1, <0.001) [27,38]. Currently, statistical measures based on colorectal tumour features (outlined below) and variant segregation are incorporated into the MMR multifactorial model as LRs [37], to ensure at least one additional piece of evidence is combined with the bioinformatically derived probabilities. Importantly, the individual components must be independent [34], thus highly correlated/overlapping information cannot be used together, such as the MSI and IHC status from the same tumour.

## 3. Tumour Features Suitable for Use in MMR Gene Variant Classification

Tumour MSI status can be used as both a positive predictor (MSI) and negative predictor (microsatellite stable—MSS) for MMR gene pathogenic variant status. Similarly loss of protein expression identified using IHC can be a positive predictor and intact protein expression a negative predictor. MSI/IHC status usually triggers MMR gene mutation screening in an index case, particularly with the implementation of universal testing for CRCs [5,43,44], thus ascertainment of the cases needs to be taken into account when using this type of tumour information in variant classification. If only unstable or tumours with loss of protein expression are tested for germline sequence variation, then only variants associated with MSI/immunoloss will be identified. However, an independent metachronous colorectal tumour or additional clinicopathological features not used in ascertainment (*BRAF*, *MLH1* promoter methylation) could be assayed in the index tumour to aid in the interpretation of uncertain variants. Likewise, tumour features of relatives known to carry the same variant may be considered. Other features of MSI colorectal tumours, such as mucinous histology, lymphocytic infiltration, or anatomic location [45,46] may also be able to contribute to MMR gene sequence variant interpretation. However, large-scale studies with detailed pathology features recorded and in-depth MMR gene testing would be required to determine LRs.

Non-independence of tumour features needs to be considered when using this information in variant classification. For example, due to the high correlation between MSI and IHC [47,48] only one of these measures should be included for an individual tumour. When MSI and IHC results are both available but discordant, the result indicating MMR deficiency should be considered as the summary result for consideration in modelling, since there are appropriate explanations for such discordance. For example, some tumours from deleterious missense variant carriers can be MSI and have immunostable protein expression [49,50]. In contrast, sometimes IHC can be a better predictor of MMR deficiency, e.g., especially for *MSH6* pathogenic variant carriers. The latter occurs particularly when the Bethesda/National Cancer Institute (NCI) panel [51], which contains mononucleotide and dinucleotide markers, is used to test MSI. MSH6 as part of the MutSα heterodimer is primarily involved in the repair of mismatches and single nucleotide IDLs [52]. Accordingly, MSI analysis using a quasimonomorphic mononucleotide microsatellite marker panel has been shown to be more effective at identifying *MSH6* pathogenic variant carriers [53], and demonstrates superior performance compared to the NCI panel [54,55].

Both the mononucleotide and NCI panels are currently used to detect MSI in tumours. These microsatellite marker panels have subsequently been used to test for MSI in extracolonic tumours [53,56,57,58], but were originally developed to distinguish MSI and MSS colorectal tumours. Presence of MSI (and loss of protein expression) can also be used to characterize MMR deficiency in the extracolonic Lynch spectrum tumours. As the risk of endometrial cancer can be as high as CRC in Lynch syndrome [59,60,61,62,63,64,65], incorporating endometrial tumour features would increase information available for classifying MMR gene sequence variants. It has been demonstrated that there are MSI events shared between endometrial and colorectal tumours (and specific to tumour type) leading to the development of more informative mononucleotide marker panels [55,66]. Widespread use of these and similar tumour-generic MSI panels will produce a more reliable measure of MSI status in both tumour types and more data to derive tumour-generic LRs for the quantitative model.

MSI is also detected in ~15% of spontaneous types of both colorectal and endometrial tumours [8,67,68]. Accordingly, colorectal tumour *BRAF* V600E status, or colorectal/endometrial tumour *MLH1* promoter methylation (that are rarely detected in Lynch Syndrome associated tumours [21,69]), are very informative for identifying probable non-causal variants among mismatch-repair-deficient tumours. The successful application of IHC for identification of BRAF V600E [70,71] makes it more readily utilized in molecular and genetic testing algorithms. However, *MLH1* promoter methylation has been shown to be superior to *BRAF* as a negative predictor in colorectal tumours [72], and the main indicator of non-pathogenicity in endometrial tumours where *BRAF* V600E status has no predictive value at all [21]. Thus, *MLH1* methylation would appear to be the better tumour test to pursue in the clinical setting.

Acquired *MLH1* promoter hypermethylation is also frequent in other MMR gene mutation-negative MSI Lynch spectrum tumours: Ovarian [73,74], gastric [75] and urothelial tumours [76]. Thus, there is value in using large studies of other Lynch spectrum cancer to explore the positive or negative predictive value of tumour features of additional LS cancers for interpreting the clinical significance of MMR gene variants.

With the recent technological advances in tumour sequencing, the hypermutated molecular tumour signature [55,77,78] could be used as an alternative to MSI testing to identify MMR deficient tumours. Additionally, acquired events in both alleles of an MMR gene (pathogenic mutation or loss of heterozygosity) have been demonstrated to cause MMR deficiency in more than 50% of colorectal and endometrial tumours without a germline MMR gene mutation or *MLH1* promoter methylation [24,25]. Therefore, there is potential value as a predictor for *in vivo* loss of function, e.g., if a variant is also seen as a somatic second hit with a known pathogenic mutation in a tumour with IHC loss or MSI then that could be seen as equivalent to a functional assay.

## 4. Qualitative and Quantitative Use of MSI in MMR Gene Variant Classification

**Figure 2:**
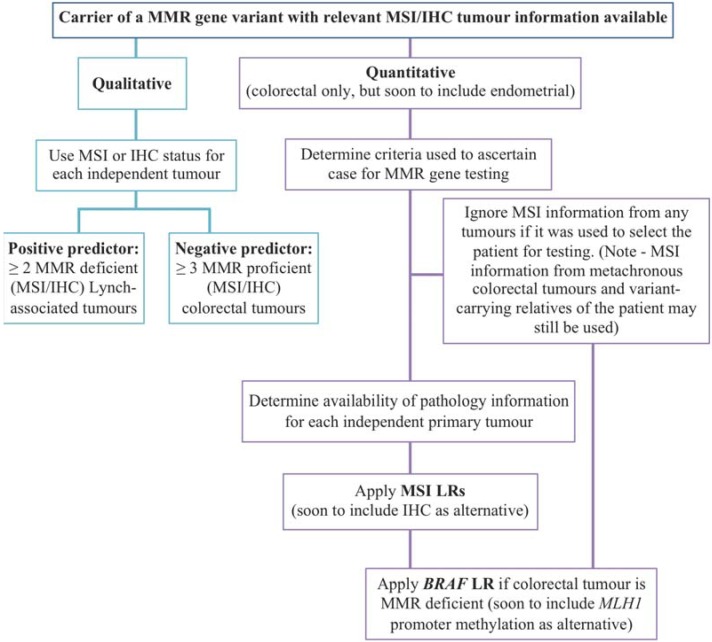
Flowchart of variant classification process using tumour features. MMR—mismatch repair; MSI—microsatellite instability; IHC—immunohistochemistry; LR—likelihood ratio.

The quantitative approach (multifactorial likelihood model) is the preferable one for MMR gene variant classification, because the final output is a quantitative probability of pathogenicity. The model currently includes LRs already calculated for MSI together with *BRAF* tumour status [37], and pending further analysis of datasets it is quite feasible to implement other tumour features mentioned in the previous section. For use in the interim, qualitative experience-based criteria have been developed to ensure that as much information could be used to aid in variant classification. Figure 2 outlines the process of variant classification using tumour characteristics, such as MSI.

In the InSiGHT qualitative rules, two or more independent MMR deficient tumours are required to contribute to classification of a MMR variant as pathogenic [27]. This can include multiple primary tumours from a single individual (as long as they are proven metachronous). The need for multiple tumour results for carriers of a given variant is implemented to minimize the chance of acquired somatic changes, such as *MLH1* promoter methylation and now biallelic somatic mutations [24,25], being the cause of tumour MMR deficiency rather than the presence of a pathogenic mutation. This strategy also in some way minimizes mis-classification due to potential technical issues and the issues of poor sensitivity of MSI reported for *MSH6* variants, mentioned above.

Few studies have addressed using MSI/IHC in a statistical approach for classification of MMR variants of uncertain clinical significance. The first report of a Bayesian integrated approach to incorporate MSI/IHC [79] included an LR for clinicopathologic features (IHC/MSI) derived using a large dataset [80]. However, ascertainment bias was not taken into account, in that only MSI tumours were tested for germline variants in the reference set, leading to overestimation of the likelihood of pathogenicity with an OR of ~27:1. Whereas, in a previous study we derived LRs for colorectal tumour MSI and *BRAF* V600E status using a large and well-characterised dataset (where testing for germline alterations was performed on a random subset of MSS tumours), and applied them carefully considering issues relating to ascertainment bias [37]. These analyses demonstrated that a MMR gene variant present in an MSI-H colorectal tumour has ~7–9-fold chance of being disease-causing, and if also wild-type *BRAF* the likelihood of being disease-causing increases to 12 to13-fold. This LR has since been used in additional studies [27,81]. The measures of LS-associated tumour features played an integral part in the categorization of over 60% MMR gene variants analysed using multifactorial likelihood analysis into classes associated with management recommendations (*i.e.*, classes 1, 2, 4 and 5).

In the future it would be beneficial if a population-based study could be used to estimate statistical measures for tumour features (*i.e.*, IHC and *MLH1* promoter methylation) of endometrial tumours [8]. If MSI were to be incorporated into the model for endometrial tumours, a mononucleotide marker panel would be more suitable than the NCI panel for testing endometrial cases [55,82].

## 5. Conclusions

Tumour MSI and IHC status can be very useful in aiding in the interpretation of MMR gene variants of uncertain clinical significance. Patient ascertainment for gene testing on the basis of tumour phenotype can limit their usefulness in multifactorial likelihood analyses and could lead to misinterpretation of variant pathogenicity. As the cost of panel gene testing for cancer predisposition falls and the perceived utility of testing multiple genes in parallel increases, there may well be a move to a universal testing system where essentially all colon (and possibly endometrial) cancer cases get a panel test prior to any testing for tumour features. For the purpose of utility of tumour features for multifactorial modelling, subjects would no longer be ascertained based on personal/family history and/or MSI/IHC. Thus, ascertainment-based confounding would be reduced or even removed. The appropriate tumour test could then become a reflex assay and the result used to help evaluate unclassified variants. In the interim, tumour MSI (and IHC) status can still be a useful tool, in combination with other clinical, molecular, and functional assay information, to aid in the interpretation of MMR gene variants of uncertain clinical significance.
